# Collaboration Networks, Trends, and Scientific Impact in Avoidable Mortality Research (2000–2024): A Bibliometric Study

**DOI:** 10.1155/bmri/1855456

**Published:** 2026-05-21

**Authors:** Miguel Paco-Fernández, Marysela Ladera-Castañeda, Daysi Diaz-Obregón, Luis Cervantes-Ganoza, César Cayo-Rojas

**Affiliations:** ^1^ Research Directorate, Institute for Health Technology Assessment and Research (IETSI), Social Health Insurance of Peru (EsSalud), Lima, Peru; ^2^ School of Stomatology, Universidad Privada San Juan Bautista, Lima, Peru, upsjb.edu.pe

**Keywords:** amenable mortality, avoidable mortality, bibliometrics, preventable mortality, treatable mortality

## Abstract

**Background:**

This study is aimed at conducting a bibliometric analysis of avoidable mortality from 2000 to 2024 to identify collaboration networks, research trends, and scientific impact in this field.

**Methods:**

A bibliometric, descriptive, and retrospective study was conducted using the Scopus Advanced Search interface, controlled vocabulary related to avoidable mortality, and Boolean operators (“AND” and “OR”). Analyses were performed in R (v4.3.1) using the bibliometrix/biblioshiny package and included indicators of publication volume, fractional authorship, total normalized citations, citation counts, and the *h*‐, *g*‐, and *m*‐indexes. International collaboration was assessed using coauthorship indicators and collaboration networks. Bradford′s law was applied to identify core journals. In addition, keyword co‐occurrence networks and thematic temporal evolution were examined to characterize research fronts and shifts over time.

**Results:**

A total of 3031 documents from 1450 journals were retrieved, with an annual growth rate of 9%. On average, articles received 27.8 citations, totaling approximately 84,000 citations overall. Authors had an average of 5.2 coauthors per article, and 244 single‐authored papers were identified. International coauthorship accounted for 23.8% of publications. The United States and the United Kingdom published most frequently in PLOS ONE and BMJ Open. Coauthorship networks revealed weak intergroup connections, with international collaboration concentrated along the US–Western Europe axis. Brazil and South Africa emerged as key regional nodes in the Global South. The thematic focus evolved from prevention‐ and health‐service–related topics to population profiles and injury, and more recently to COVID‐19–related terminology and pregnancy.

**Conclusion:**

This bibliometric analysis demonstrates a rising interest in avoidable mortality, reflected in increased scientific output and limited intergroup connectivity within international collaboration networks. Only a limited number of authors consistently contribute impactful research in both general and specialized journals. These findings highlight the importance of fostering collaborative, high‐quality translational research to inform public policy and address the regional determinants of avoidable mortality, particularly in the Global South.

## 1. Background

Avoidable mortality is recognized as a fundamental metric for evaluating health system performance and detecting inequalities. It comprises preventable and treatable components and, within the harmonized OECD–Eurostat framework, is generally assessed for deaths occurring before the age of 75 [1, 2]. This approach, rooted in the classic literature on sentinel health events and the measurement of the quality of medical care [3], has been incorporated into international comparisons and has gained renewed prominence following the disruption caused by the COVID‐19 pandemic, which interrupted progress and exposed disparities between countries and social groups [4]. Conceptually, this framework aligns with Sustainable Development Goal (SDG) Target 3.4, which is aimed at reducing premature mortality from noncommunicable diseases through prevention and treatment, underscoring its policy relevance for guiding health system priorities [5, 6].

The evolution of this construct began with the concept of “sentinel health events,” originally proposed as a clinical approach to measuring the quality of medical care rather than as a generic indicator of healthcare failure [7]. It then progressed to international comparisons of amenable mortality that linked these deaths to healthcare performance [8] and was later strengthened by empirical validation studies (e.g., AMIEHS), which identified a core set of causes with reproducible criteria [9]. Analytically, this approach requires conceptualizing avoidable mortality as an operational construct, involving explicit decisions on cause selection, age thresholds, age standardization methods, data quality, and inclusion or exclusion of external causes. These decisions should align with the OECD–Eurostat lists and the methodological guidelines of the UK Office for National Statistics (ONS) [1, 6, 10, 11]. In practice, statistical agencies such as the ONS have developed procedures to ensure temporal consistency and minimize coding biases, providing a robust methodological reference framework [11].

More recently, the burden of avoidable mortality has remained substantial across high‐ and middle‐income countries [4]. In England and Wales, approximately one in five deaths in 2023 were classified as avoidable (< 75 years), with persistent socioeconomic disparities [12, 13]. Within the European Union, 1.1 million deaths in 2022 (aged < 75 years; 257.8 per 100,000 population) were considered avoidable—89.7 per 100,000 treatable and 168.1 per 100,000 preventable [14]. In Latin America, the Pan American Health Organization (PAHO) and the World Health Organization (WHO) reported an adjusted rate of potentially avoidable premature mortality of 139.1 per 100,000 in Chile in 2019, representing a 30.6% reduction since 2000. Of these, 79.1 per 100,000 were preventable and 60 per 100,000 were treatable—below the regional average of 89.6 [15]. In Mexico, a national analysis from 2000 to 2019 revealed only a modest decline (297 → 281 per 100,000), with 170 per 100,000 preventable and 111 per 100,000 treatable deaths, and notable subnational and sex‐related disparities [16]. These contextual differences illustrate the value of bibliometric mapping (e.g., Scopus) for identifying thematic foci, research trajectories, and evidence gaps related to “avoidable mortality” [17, 18]. Existing bibliometric studies have tended to focus on broader constructs such as premature mortality or on specific disease areas, rather than on avoidable mortality as a unified analytic field. Although such studies are useful for understanding broad mortality patterns, they do not specifically capture the dual public health and health care dimensions that define avoidable mortality within established operational frameworks. In parallel, recent conceptual and scoping literature has emphasized that avoidable mortality remains a heterogeneous field in terms of cause lists, age thresholds, and policy interpretation, which further justifies the need for a focused bibliometric mapping.

Despite its conceptual consolidation and widespread use in international comparisons [1, 4–6], the literature still lacks a comprehensive global bibliometric mapping focused specifically on avoidable mortality as a distinct analytic construct over an extended time horizon (2000–2024). Existing studies have primarily addressed premature mortality, specific clinical domains, or national and subnational analyses [12–16, 19–22]. A related bibliometric analysis examined global research activity on premature mortality using Web of Science [23]; however, premature mortality is a broader construct centered on early death irrespective of whether the death was preventable or treatable. By contrast, avoidable mortality explicitly integrates preventable and treatable components within operational frameworks used to assess health‐system performance, which justifies a separate bibliometric mapping rather than a merely complementary extension of the premature‐mortality literature. Therefore, rather than duplicating broader mortality mappings, the present study seeks to characterize the global scientific production, intellectual structure, thematic evolution, and collaboration networks specifically associated with avoidable mortality. Accordingly, the aim of this study was to conduct a bibliometric analysis of avoidable mortality between 2000 and 2024 to identify collaboration networks, research trends, and the impact of scientific output in this domain.

## 2. Methods

### 2.1. Study Design

This study employed a bibliometric, descriptive, and retrospective design. The study covered publications from January 1, 2000, to December 31, 2024.

### 2.2. Search Strategy

Data were extracted from Scopus and citation metrics were recorded on September 11, 2025. The Scopus database (Elsevier, United States) was accessed via the Advanced Search interface. Search parameters included Boolean operators (“AND” and “OR”), document type, and time range filters. A total of 3031 documents were retrieved. Scopus was selected as the source database because it provides broad multidisciplinary coverage, standardized bibliographic metadata, and direct compatibility with bibliometrix/biblioshiny workflows for science mapping. To improve transparency regarding search completeness, the search equation was checked against key relevant articles in the field. However, the exclusive use of Scopus may have led to the omission of records indexed only in Web of Science, PubMed, or regional databases, particularly those from underrepresented settings. No language filter was applied in Scopus. Beyond the English, Spanish, and Portuguese terms included in TITLE‐ABS‐KEY, retrieval of records in other languages relied on Scopus′ normalization of Index Keywords into English; consequently, articles published in other languages lacking index keywords in English may not have been captured by the search equation. This decision prioritized coverage without compromising search reproducibility.

The exact Scopus query was as follows: (TITLE‐ABS‐KEY (“avoidable mortality” OR “amenable mortality” OR “treatable mortality” OR “preventable mortality” OR “mortality amenable to health care” OR “mortality amenable to healthcare” OR “avoidable deaths” OR “amenable deaths” OR “preventable deaths” OR “muertes evitables” OR “óbitos evitáveis” OR “obitos evitaveis”)) AND PUBYEAR >1999 AND PUBYEAR <2025 AND (EXCLUDE (EXACTKEYWORD,“Animals”) OR EXCLUDE (EXACTKEYWORD,“Animal”) OR EXCLUDE (EXACTKEYWORD,“Animal Model”) OR EXCLUDE (EXACTKEYWORD,“Nonhuman”)) AND (LIMIT‐TO (SRCTYPE,“j”)) AND (LIMIT‐TO (DOCTYPE,“ar”)) AND (LIMIT‐TO (PUBSTAGE,“final”)).

### 2.3. Selection Criteria

Records were included if they were original research articles from Scopus‐indexed journals, published in their final stage between January 1, 2000, and December 31, 2024, with no language restrictions, and containing at least one of the predefined terms for avoidable mortality in the TITLE‐ABS‐KEY fields. Records tagged with controlled descriptors related to animals were excluded. Because these eligibility criteria were embedded directly in the Scopus query, the 3031 retrieved records constituted the final analytical corpus.

### 2.4. Procedure in Bibliometrix

All records were exported from Scopus in CSV format and analyzed in R (RStudio) using bibliometrix/biblioshiny, following a science‐mapping workflow that included data collection, performance analysis, network construction, visualization, and interpretation. Before analysis, metadata were reviewed and cleaned to improve consistency, including standardization of author names and harmonization of duplicated or variant entries when necessary. The corpus was generated by running the predefined Scopus query and applying the prespecified eligibility criteria embedded in the search strategy. The resulting dataset was then imported into R for bibliometric analyses, including source, author, country, and keyword analyses, as well as network visualizations used to support the interpretation of research output patterns and thematic structure. All analyses were conducted through the biblioshiny graphical interface of the bibliometrix package in R, and no custom R script was used. To support reproducibility, the full search query, eligibility criteria, bibliometric workflow, counting methods, normalization approach, clustering algorithm, and analytic settings applied in biblioshiny are reported in the manuscript. In addition, the stoplist used for the thematic analyses is provided as Supporting Information.

### 2.5. Data Analysis

Bibliometric networks were normalized using association strength. Coauthorship networks for authors and countries were constructed using fractional counting. For the thematic analyses, Scopus Index Keywords were used with full counting, and a conservative stoplist was applied to improve thematic interpretability. Visualization‐specific thresholds and graphical parameters are reported in the corresponding figure legends. Community detection was performed using the Louvain algorithm. Performance indicators were estimated, including annual research output and growth, and productivity by sources (journals), authors, countries, and institutions. Impact was assessed using total citations (TC), citations per year per document, and the *h*‐, *g*‐, and *m*‐indexes at the author, country, and source levels. Collaboration was characterized using the collaboration index, national versus international coauthorship rates, and coauthorship network structures. Bradford′s law was applied to identify core journals, and Lotka′s law was used to evaluate author productivity [24, 25].

### 2.6. Ethical Considerations

As this study was based exclusively on the secondary analysis of bibliometric metadata obtained from the Scopus database, it did not require ethical approval from an institutional review board.

## 3. Results

Between 2000 and 2024, a total of 3031 original research articles were published across 1450 journals. Annual scientific output showed a 9.0% growth rate. On average, articles received 27.8 citations, with a total of approximately 84,000 citations across the corpus. The mean number of coauthors per article was 5.2, whereas 244 single‐authored papers were identified. International coauthorship accounted for 23.8% of the total, reflecting a moderate level of global collaboration. Thematic breadth was high, with 13,591 author keywords and 9631 indexed keywords recorded in Scopus (Table 1).

**Table 1:** Main characteristics of the analyzed articles.

Indicator	Results
Time period (years)	2000–2024
Sources (journals)	1450
Documents	3031
Annual growth rate (%)	9.0
Average document age (years)	8.5
Average citations per document	27.8
References	22,478
Indexed keywords	9631
Author keywords	13,591
Authors	13,015
Single‐authored documents	244
Coauthorship per document	5.2
International coauthorship rate	23.8%

**Figure 1:**
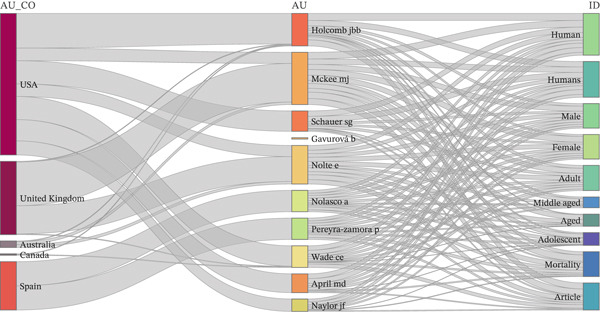
Three‐field plot showing the relationships between countries, authors, and indexed keywords in Scopus from 2000 to 2024.

The three‐field plot revealed that scientific output on avoidable mortality was concentrated primarily in the United States, followed by the United Kingdom. These countries were closely associated with a small core of authors (*Holcomb J.*, *McKee M.*, *Schauer S.*, *Gavurová B.*, *and April M.*). The most frequent indexed keywords were population descriptors, highlighting the predominance of an epidemiological focus (Figure 1).

The top 10 journals ranked by *h*‐index identified the *Journal of Trauma* as the leading source (*h* = 24), followed by *PLOS ONE* (*h* = 20) and *BMC Public Health* (*h* = 19), indicating that these journals form the core of consistently cited publications on avoidable mortality from 2000 to 2024. Similarly, *PLOS ONE* ranked first in publication volume (*N* *P* = 58), followed by *BMC Public Health* (*N* *P* = 39). In addition, these two journals were distinguished by their highly cited papers (*g* = 38 and *g* = 39, respectively). *The Journal of Trauma and Acute Care Surgery* and *PLOS ONE* showed the highest recent impact (*m* = 1.214 and *m* = 1.111, respectively) (Table 2).

**Table 2:** Volume and citation impact of the top 10 journals ranked by *h*‐index in avoidable mortality research.

Journal	Country	*h*‐index	*g*‐index	*m*‐index	TC	NP	PY‐start
Journal of Trauma∗	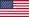	24	26	0.960	2234	26	2001
PLOS ONE ^(OA)^	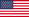	20	38	1.111	1494	58	2008
BMC Public Health ^(OA)^	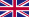	19	39	0.826	1589	39	2003
Journal of Epidemiology and Community Health	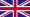	17	25	0.654	960	25	2000
Journal of Trauma and Acute Care Surgery	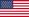	17	29	1.214	869	30	2012
Injury	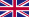	15	26	0.625	697	30	2002
Social Science & Medicine	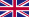	14	22	0.538	849	22	2000
BMJ Open ^(OA)^	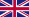	13	25	0.929	687	40	2012
International Journal of Environmental Research and Public Health ^(OA)^	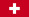	13	19	0.765	422	33	2009
Prehospital Emergency Care	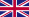	12	17	0.545	368	17	2004

*Note:* Asterisk “∗” denotes discontinued in 2011.

Abbreviations: *g*‐index, gives greater weight to highly cited papers (captures “hits”); *h*‐index, number of papers with at least that number of citations (reflects the base of consistently cited work); *m*‐index, *h*‐index normalized by years since PY‐start (reflects speed of citation impact accumulation); NP, number of papers on the topic published by the journal; OA, Open Access journal; PY‐start, first year the journal appears in the dataset for this topic; TC, total citations received by those papers.

**Figure 2:**
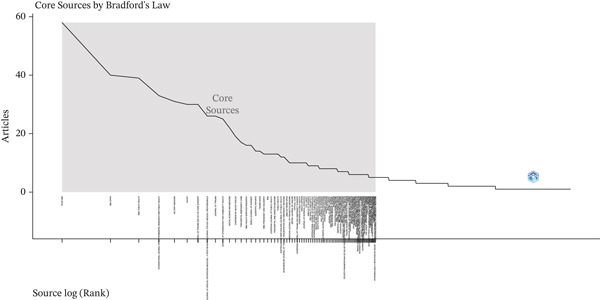
Distribution of journals according to Bradford′s law.

According to Bradford′s Law, a small core of journals tends to concentrate a disproportionate share of publications on a given topic. In the present study, Zone 1 (the core) included a small number of journals that concentrated approximately one‐third of all articles. The most prominent journals in this zone were *PLOS ONE* (58 articles) and *BMJ Open* (40 articles), which served as the main dissemination channels for research on avoidable mortality. Zone 2 consisted of a larger number of journals, each publishing between two and five articles, including *Epidemiologia e Serviços de Saúde*: *Revista do Sistema Único de Saúde do Brasil* and the *European Journal of Emergency Medicine*. Finally, Zone 3 exhibited a long‐tail distribution, with one or two articles per journal—for instance, *Systematic Reviews* and *Systems*—reflecting isolated or highly specific contributions to the topic (Figure 2).

**Figure 3:**
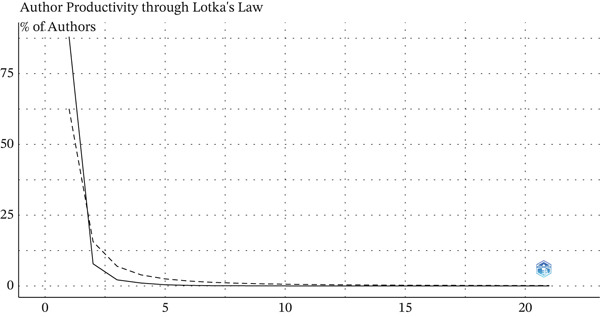
Author scientific output according to Lotka′s law.

According to Lotka′s Law, most authors tend to contribute only one publication, whereas a much smaller group accounts for repeated productivity. In the present study, 88% of all authors (*n* = 11,455) contributed a single article, whereas only 0.95% (*n* = 123) published five or more. In addition, the dashed line representing Lotka′s theoretical model lay above the observed distribution for authors with two to three publications, indicating that there were fewer highly productive authors than predicted by the classical model (*α* > 2). This suggests that the bulk of scientific output on avoidable mortality is driven by a large number of authors with occasional contributions. Conversely, a small group of recurring authors has sustained ongoing research in this field (Figure 3).

Among the top 10 most productive authors, *Holcomb J.* (*h* = 16, *g* = 21) and *McKee M.* (*h* = 15, *g* = 18) had the highest average citation rates (approximately 80 and 104 citations per article, respectively). In contrast, *Schauer S.* and *April M.* recorded the highest *m*‐index values (*m* = 1.25), followed by *Naylor J.* (*m* = 1.00), indicating a rapid accumulation of citations per year since their first publication on avoidable mortality (2018). Finally, the fractional count (NP/AF) showed average collaboration levels of six to eight coauthors per article for *Wade C.*, *Nolasco A.*, and *Pereyra-Zamora P.*, and two to four coauthors per article for *Gavurová B.*, *McKee M.*, and *Nolte E*. (Table 3).

**Table 3:** Top 10 authors ranked by productivity, citation impact, and collaboration.

Author	Affiliation	Country	*h* *-*index	*g* *-*index	*m* *-*index	TC	NP	PY‐start	AF
Holcomb J.B.B.	UAB Center for Injury Science	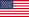	16	21	0.84	1681	21	2007	3.43
Schauer S.G.	U.S. Army Medical Center of Excellence	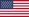	10	15	1.25	260	21	2018	3.38
April M.D.	Brooke Army Medical Center	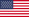	10	15	1.25	239	18	2018	2.82
McKee M.J.	London School of Hygiene & Tropical Medicine	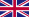	15	18	0.65	1864	18	2003	4.81
Gavurová B.	Technická Univerzita v Košiciach	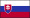	6	9	0.55	97	13	2015	4.53
Nolte E.	London School of Hygiene & Tropical Medicine	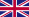	10	12	0.44	1723	12	2003	3.88
Wade C.E.	McGovern Medical School	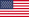	10	12	0.53	545	12	2007	1.55
Naylor J.F.	Madigan Army Medical Center	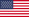	8	11	1.00	182	11	2018	1.76
Nolasco A.	Universitat d’Alacant	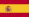	7	10	0.39	157	10	2008	1.40
Pereyra‐Zamora P.	Universitat d’Alacant	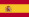	7	10	0.39	157	10	2008	1.40

Abbreviations: AF, fractional authorship credit per paper; *g*‐index, gives greater weight to highly cited papers (detects “hits”); *h*‐index, number of papers with at least that number of citations (reflects the base of well‐cited publications); *m*‐index, *h*‐index normalized by years since PY‐start (indicates the speed at which impact accumulates in this topic); NP, number of papers on the topic published by the author; PY‐start, first year the author appears in the dataset for this topic;TC, total citations received by those papers.

**Figure 4:**
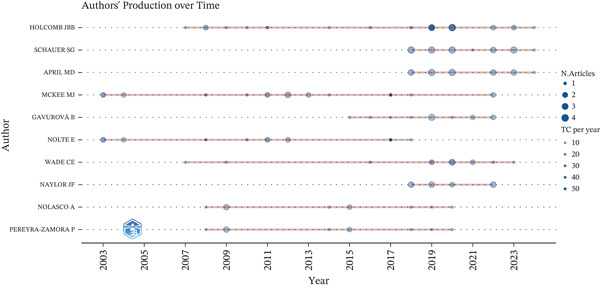
Annual scientific output of the 10 most productive authors. TC, total citation.

The authors with the highest scientific output on avoidable mortality showed increased productivity between 2018 and 2021, reaching a peak of three to four papers per year. *Holcomb J.*, *Schauer S.*, and *April M.* recorded the highest annual citation rates. *McKee M.* and *Nolte E.* were the earliest contributors to research on avoidable mortality, beginning in 2003. In addition, several authors showed intermittent publication trajectories before 2018, followed by recent peaks in both productivity and impact (Figure 4).

Among the 10 most cited articles, the studies by *Walker* (2015) with 2348 citations, *Foreman* (2018) with 1993 citations, and *Gould* (2012) with 1936 citations stood out. When adjusted for publication age, the leading papers were those by *Foreman* with 249.13 citations per year, *Perin* with 213.75 citations per year, and *Walker* with 213.45 citations per year. Finally, according to normalized TC, *Perin* (58.90), *Walker* (48.35), and *Foreman* (45.51) ranked highest. These results indicate a recent, crosscutting impact across both general and specialty journals (Table 4).

**Table 4:** Top 10 most influential articles.

Authors (year)	Country	Journal	DOI	TC	TC per year	Normalized TC
Walker et al. (2015) [26]	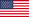	*JAMA Psychiatry*	10.1001/jamapsychiatry.2014.2502	2348	213.45	48.35
Foreman et al. (2018) [27]	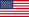	*The Lancet*	10.1016/S0140‐6736(18)31694‐5	1993	249.13	45.51
Gould et al. (2012) [28]	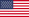	*CHEST*	10.1378/chest.11‐2297	1936	138.29	37.53
Thorgeirsson et al. (2008) [29]	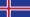	*Nature*	10.1038/nature06846	1287	71.50	18.98
Spahn et al. (2019) [30]	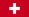	*Critical Care*	10.1186/s13054‐019‐2347‐3	1013	144.71	31.65
Coleman (2011) [31]	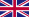	*The Lancet*	10.1016/S0140‐6736(10)62231‐3	1009	67.27	15.40
Umscheid et al. (2011) [32]	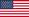	*Infection Control & Hospital Epidemiology*	10.1086/657912	916	61.07	13.98
Perin et al. (2022) [33]	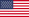	*The Lancet Child & Adolescent Health*	10.1016/S2352‐4642(21)00311‐4	855	213.75	58.90
Cook et al. (2011) [34]	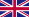	*British Journal of Anaesthesia*	10.1093/bja/aer059	787	52.47	12.01
Balakrishnan et al. (2019) [35]	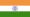	*The Lancet Planetary Health*	10.1016/S2542‐5196(18)30261‐4	716	102.29	22.37

Abbreviation: TC, total citations.

**Figure 5:**
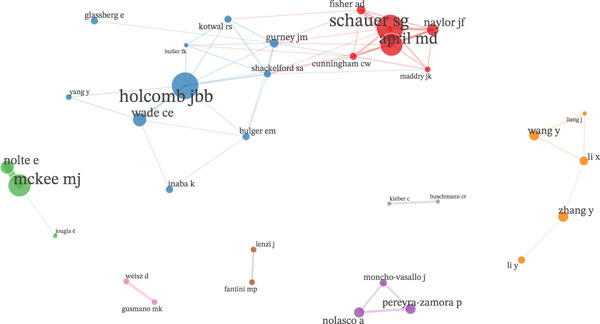
Coauthorship collaborative networks. The author collaboration network was generated in biblioshiny using fractional counting, association normalization, automatic layout, and the Louvain clustering algorithm. The visualization was limited to 50 nodes, with a repulsion force of 0.5; isolated nodes were removed, and links with a minimum of one edge were retained. Nodes represent authors, and connecting lines represent coauthorship links.

Collaborative networks on avoidable mortality showed a predominant collaborative structure composed of two clearly connected subclusters centered on Holcomb J.B.B. and on Schauer S.G./April M.D., respectively, both largely linked to trauma and emergency medicine. Several additional clusters were smaller and only weakly connected to the main structure, suggesting specialization within research teams and relatively limited exchange between groups (Figure 5).

**Figure 6:**
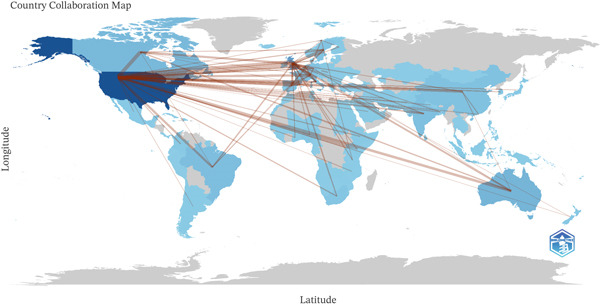
Global collaboration network between countries. Only links meeting a minimum edge threshold of five were displayed. Edge size was set to five for graphical visualization. Nodes represent countries, and connecting lines represent international coauthorship links between them.

The collaboration map reveals the United States as the central hub of international collaboration, with strong connections to Western Europe. A dense collaborative network is evident between the United Kingdom and European Union countries (Spain, Italy, Germany, France, and the Netherlands), with Australia and several Asian countries serving as secondary bridging nodes. In Latin America, Brazil serves as the main connection point with the United States and Europe, followed by Mexico, Colombia, and Argentina. In Africa, South Africa occupies a leading position, maintaining active collaborations with the United States and Europe (Figure 6).

**Figure 7:**
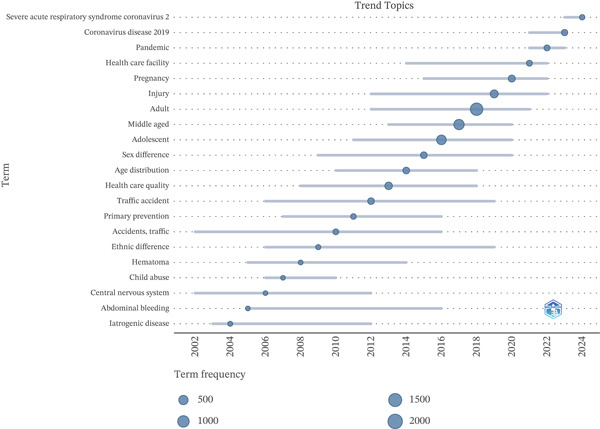
Temporal evolution of topics related to avoidable mortality. Trend topics were generated in biblioshiny using the Scopus Index Keywords field, with a minimum word frequency of five and one word displayed per year. A conservative stoplist was applied to remove generic methodological, geographic, and nonsubstantive indexing terms; the full stoplist is provided in the Supporting Information. Dot size represents term frequency, and horizontal lines indicate the time span during which each term appeared in the dataset. The visible time span and selected terms reflect the bibliometrix visualization settings and therefore do not necessarily represent all topics across the full 2000–2024 study period.

The temporal mapping of trending topics identified three broad stages. Until approximately 2013, the most visible terms were related to prevention and healthcare performance, including “traffic accident,” “health care quality,” and “primary prevention.” Between 2014 and 2019, the thematic profile shifted toward population descriptors and injury‐related terminology, with terms such as “adult,” “middle‐aged,” “adolescent,” and “injury” becoming more prominent. From 2020 onwards, COVID‐19–related terminology (“coronavirus disease 2019,” “pandemic,” and “severe acute respiratory syndrome coronavirus 2”) became dominant and was accompanied by “pregnancy.” Overall, the map suggests a transition from prevention‐ and quality‐oriented themes to population and injury profiles, followed by postpandemic topics centered on COVID‐19–related terminology, with pregnancy emerging as a visible accompanying theme (Figure 7).

**Figure 8:**
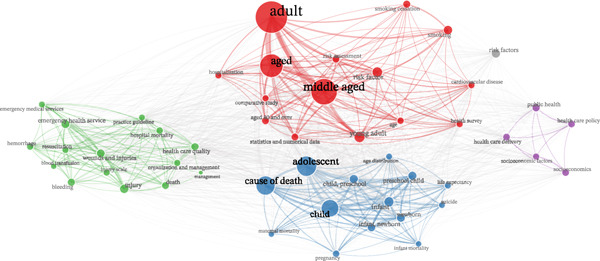
Keyword co‐occurrence map based on Scopus Index Keywords. The network was generated in biblioshiny using association normalization, automatic layout, and the Louvain clustering algorithm. The visualization was limited to 50 nodes, with a repulsion force of 0.5; isolated nodes were removed, and only terms with a minimum of two edges were retained. A conservative stoplist was applied to remove generic methodological, geographic, and nonsubstantive indexing terms; the same stoplist is provided in the Supporting Information used for the trend‐topics analysis. Node size represents keyword frequency, spatial proximity reflects the strength of co‐occurrence between terms, and colors indicate clusters identified by the Louvain algorithm.

The keyword co‐occurrence map revealed a four‐cluster thematic structure in the avoidable mortality literature. The red cluster was dominated by adult population terms (e.g., adult, aged, middle‐aged, and young adult) and was closely connected to risk‐related and chronic disease descriptors such as smoking, smoking cessation, risk assessment, risk factor, cardiovascular disease, and health survey, suggesting a strong emphasis on behavioral and epidemiologic determinants in adult mortality research. The blue cluster grouped pediatric and life‐course terms (e.g., infant, child, adolescent, preschool child, newborn, infant mortality, maternal mortality, pregnancy, life expectancy, and suicide), indicating a second thematic domain centered on early‐life and population mortality outcomes. The green cluster concentrated on emergency and hospital‐care processes (e.g., emergency medical services, emergency health service, resuscitation, hemorrhage, bleeding, blood transfusion, wounds and injuries, injury scale, hospital mortality, health care quality, and organization and management), highlighting the role of timely clinical response and service organization in reducing fatal outcomes. A smaller purple cluster linked public health, health care policy, health care delivery, socioeconomic factors, and socioeconomics, reflecting a complementary policy and social determinants perspective. Overall, the network suggests that avoidable mortality research is structured around adult risk profiles, early‐life mortality, emergency‐care management, and broader policy and socioeconomic frameworks (Figure 8).

## 4. Discussion

The aim of this study was to perform a bibliometric analysis of avoidable mortality in humans between 2000 and 2024 to identify collaborative networks, research trends, and the impact of scientific production in this field. The findings revealed a steady global expansion of research on avoidable mortality during this period, with 3031 original articles published across 1450 journals, reflecting an annual growth rate of 9%. Nearly one quarter of the total output involved international coauthorship. Scientific production was primarily concentrated in countries such as the United States and the United Kingdom, whereas the editorial core was led by *PLOS ONE* and *BMJ Open*. From a clinical and health‐systems perspective, mapping the evolution of avoidable mortality research is relevant because it helps identify where evidence has concentrated (and where it remains scarce) for conditions and care pathways in which timely, effective interventions can reduce premature deaths, thereby informing priorities for prevention, service delivery, and quality improvement. The prominence of a small set of high‐throughput, generalist journals likely reflects the field′s cross‐disciplinary nature and the demand for rapidly disseminating evidence relevant to policy and service delivery; nevertheless, journal‐level patterns should be interpreted descriptively and complemented by article‐level appraisal. The vast majority of authors contributed a single paper on avoidable mortality, and collaborative networks showed limited intergroup connectivity. Thematic evolution progressed from prevention and health service performance to population profiles and injury‐related terminology, and more recently to COVID‐19–related terminology accompanied by pregnancy.

With regard to the profile of the most cited research, globally scoped studies were predominantly published in multidisciplinary, general medicine, and specialty journals [26–35]. These works focused on international surveillance and comparability, for example, studies on prognostic estimates and comparable series of mortality and life expectancy [27], as well as analyses of causes of death in children under 5 years of age [33]. Their impact can likely be attributed to the speed with which they accumulate citations, given their value for policy planning and goal monitoring. In addition, publications presenting clinical guidelines, consensus statements, and audits with immediate applicability showed high impact—for instance, recommendations on venous thromboembolism (VTE) prophylaxis in nonorthopedic surgery [28], the management of major bleeding in trauma [30], and airway complications [34]. Likewise, studies addressing preventable risks—such as healthcare‐associated infections [32] and air pollution in India [35]—and major determinants—such as tobacco and genetic factors [29], or international oncology performance [31]—also exerted strong influence, closely aligned with the avoidable mortality research agenda. Furthermore, a meta‐analysis quantifying excess mortality in mental disorders [26] received substantial attention due to its relevance for public health policy. Overall, these patterns explain why articles published in prestigious, high‐impact journals (*The Lancet*, *Nature*, and *JAMA*), characterized by methodological rigor and results that inform the implementation of health solutions, tend to dominate recent and normalized citation metrics in the field of avoidable mortality.

The results showed sustained growth but limited international collaboration, with an average of five coauthors per paper and 244 single‐authored publications. These indicators suggest a moderate level of international coauthorship within the analyzed corpus and should be interpreted cautiously in view of field‐specific and database‐related differences. Moreover, the geographical concentration of scientific production in the United States and the United Kingdom highlights asymmetries in research interest, capacity, and editorial visibility, which should be considered when interpreting the structure of the field. From an interpretive standpoint, the observed collaborative structure has implications beyond publication counts. Limited connectivity between author clusters may hinder methodological harmonization, reduce comparability across settings, and slow the translation of evidence into shared health‐system benchmarks. This issue is particularly relevant in avoidable mortality research, where differences in cause lists, age thresholds, coding practices, and equity stratifiers can substantially affect interpretation. In addition, the concentration of visible scientific output in the United States and Western Europe likely reflects asymmetries in research infrastructure, funding availability, institutional visibility, and long‐standing participation in high‐impact publication networks. For low‐ and middle‐income countries, these findings highlight the need for multicountry collaborations, harmonized operational definitions, and comparable analytic protocols that can support benchmarking, surveillance, and policy translation across settings.

This study found that the distribution of authorship followed Lotka′s Law, highlighting a relatively small core of authors who have published extensively on avoidable mortality, while revealing a broad base of occasional contributors. In this context, *Holcomb J.* stood out for both productivity and consolidated impact, whereas *Schauer S.* and *April M.* exhibited a rapid publication pace with early citation accrual, as reflected by their high *m*‐index values. Coauthorship networks showed limited intergroup connectivity, with relatively few bridging links between clusters. However, *Schauer S.* and *April M.* served as connecting nodes, and *Holcomb J.* linked multiple coauthors, which may suggest specialization by subfields (predominantly trauma and emergency medicine) and limited cross‐field permeability, indicating restricted exchange between thematic areas. This architecture of collaborative networks may favor the continuity of research on avoidable mortality, but it could hinder methodological dissemination, as reflected in geographically restricted estimates and limited study designs. In addition, the current structure may limit thematic integration with cardiovascular and equity dimensions. Therefore, fostering interregional collaborative groups and developing standardized protocols for defining “avoidable mortality”—including causal factors, age groups, and equity variables—could enhance connectivity and improve the comparability of results in this field. In practical terms, this fragmentation may translate into heterogeneous operational definitions and limited comparability across settings; strengthening multicenter consortia and shared analytic protocols could improve external validity and accelerate translation into actionable benchmarks for health systems.

Consistent with the co‐occurrence structure, the temporal analysis suggests that the field evolved from prevention‐ and healthcare‐performance–related concerns toward population profiles and injury‐related terminology, and more recently toward COVID‐19–related topics accompanied by pregnancy. Between 2000 and 2013, terms linked to prevention and healthcare quality predominated, which is consistent with the operational framing of avoidable mortality as a measure of preventable and treatable deaths and with the methodological harmonization promoted by OECD/Eurostat and subsequently adopted by the ONS [36, 37]. Between 2014 and 2019, the thematic profile shifted toward population descriptors and injury‐related terminology, reflecting the growing visibility of trauma and emergency‐care pathways. From 2020 onwards, COVID‐19–related terminology became dominant and was accompanied by pregnancy‐related terms. This recent pattern is plausible in light of evidence showing that the pandemic disrupted essential maternal and newborn services and was associated with worsening maternal and perinatal outcomes in multiple settings [38, 39]. Taken together, these findings suggest that the most recent thematic profile of the field was shaped more clearly by pandemic‐related research and its implications for maternal care than by a dominant disease‐specific axis.

Although external epidemiological indicators often identify cardiovascular and other chronic conditions as major contributors to avoidable mortality, these causes did not emerge as dominant terms in the present bibliometric mapping. This discrepancy is methodologically plausible because epidemiological indicators quantify deaths using standardized ICD‐based lists, whereas bibliometric maps depend on article labeling, keyword co‐occurrence thresholds, and the prominence of broader crosscutting themes. Accordingly, the absence of specific causes as dominant keywords should not be interpreted as evidence of low substantive relevance, but rather as a reflection of how the literature is indexed and thematically organized [6, 18, 39]. Thus, the output of an epidemiological indicator does not necessarily translate into the presence or frequency of a corresponding keyword in the literature.

The findings support the use of avoidable mortality as an operational lens for decision‐making because the mapped literature emphasizes prevention, healthcare performance, trauma and emergency‐care pathways, and, more recently, pandemic‐related maternal‐health concerns. From a management perspective, the geographic concentration of research and the moderate but uneven pattern of international coauthorship underscore the importance of strengthening collaborations involving countries in the Global South. Similarly, harmonizing terminology, operational definitions, and analytic thresholds would improve comparability across settings. At the technical level, institutionalizing performance dashboards stratified by sex, age, territory or region, socioeconomic status, and ethnicity—accompanied by both national and subnational analyses—would be valuable and advantageous. This would strengthen collaboration and interoperability under reproducible FAIR criteria, ensuring that evidence translates into targeted resource allocation and continuous monitoring with predefined statistical thresholds, thereby accelerating responses to relevant deviations [40–42].

Among the strengths of this study is the use of strict inclusion criteria, limited to original articles in the final publication stage. In addition, the use of the Scopus database allowed for the mapping of original studies that had undergone rigorous peer review processes, thereby ensuring high‐quality scientific outputs [43]. Another strength lies in the broad overview, this study provides of the evolution of avoidable mortality over the past 25 years. Importantly, this study appears to be one of the first global bibliometric mappings focused specifically on avoidable mortality in humans as a distinct analytic construct, rather than on premature mortality more broadly [23]. Therefore, it is expected to offer a comprehensive perspective on research trends in this field and to serve as a foundation for guiding future studies.

Within the limitations, it should be acknowledged that the exclusive coverage of Scopus may fail to include regional journals and those published in languages other than English. This limitation may be particularly relevant for the representation of the Global South, as recent evidence has documented regional disparities in the journal coverage of major indexing databases, including Scopus, which may affect the visibility of research produced in Latin America, Africa, and parts of Asia [44]. Although no language filters were applied, records in other languages may not have been retrieved if Scopus did not assign English‐normalized Index Keywords to those articles. In addition, reliance on keywords may dilute the representation of specific causes within the analysis of avoidable mortality, as bibliometric metrics are sensitive to parameters such as minimum frequency, cluster resolution, and disambiguation strategies [17]. However, these limitations do not invalidate the main patterns identified. It is recommended that the results be interpreted with caution and that future studies perform sensitivity analyses using different databases, threshold variations, and thesauri guided by documented lists of causes. Furthermore, the exclusion of books and book chapters may have limited the coverage of conceptual and theoretical developments in the field; however, this decision ensured the inclusion of peer‐reviewed, comparable, and standardized sources of information.

Future studies should be aimed at bridging the gap between indicators and the scientific literature by developing standardized terminology for causes associated with avoidable mortality, thereby recovering specific causes that are often obscured by aggregated keywords [45, 46]. In addition, efforts should be made to strengthen the translation of research into practice by defining metrics that monitor the time from initial publication to real‐world adoption [47]. Finally, prospective pilot studies are recommended to evaluate the performance of dashboards and alert systems designed to identify significant deviations in preventable mortality [48].

## 5. Conclusion

This bibliometric mapping characterizes a broad and expanding field, albeit one marked by geographical, editorial, and authorship concentration. Scientific production was primarily concentrated in the United States and the United Kingdom, whereas *PLOS ONE* and *BMJ Open* served as the main dissemination channels. Authorship patterns revealed a broad base of occasional contributors supported by a small, consistent core of researchers. Moreover, coauthorship networks showed limited intergroup connectivity, with relatively weak links between clusters. International collaboration was largely organized around the United States–Western Europe axis, with Brazil and South Africa acting as regional nodes in the Southern Hemisphere. Thematically, the field evolved from a focus on prevention and healthcare‐quality–related topics to population profiles and injury, and more recently to COVID‐19–related terminology and pregnancy. It is recommended that future efforts focus on standardizing definitions and methodologies, fostering interregional collaborations across the Global South, and promoting data sharing guided by equity principles to enhance comparability and practical usefulness for public health.

## Author Contributions

M.P‐F. conceived the research idea. M.P‐F., M.L‐C., D.D‐O., L.C‐G., and C.C‐R. drafted the manuscript. C.C‐R. collected the information. M.L‐C., L.C‐G., and C.C‐R. reviewed the study methodology. M.P‐F., M.L‐C., D.D‐O., L.C‐G., and C.C‐R. contributed to the interpretation of the findings.

## Funding

No funding was received for this manuscript.

## Disclosure

All authors read and approved the final manuscript. All authors critically revised the manuscript, approved the final version, and agreed to be accountable for all aspects of the work.

## Ethics Statement

As this study was based exclusively on the secondary analysis of bibliometric metadata obtained from the Scopus database, it did not require ethical approval from an institutional review board.

## Conflicts of Interest

The authors declare no conflicts of interest.

## Supporting information


**Supporting Information** Additional supporting information can be found online in the Supporting Information section. Supporting Information: stoplist_avoidable_mortality_v7. This file contains the stoplist used for the thematic analyses of avoidable mortality. The stoplist includes generic documentary, methodological, study‐design, indexing, population, epidemiological, statistical, outcome, follow‐up, geographic, organizational, and nonsubstantive terms that were removed from the Scopus Index Keywords field before generating the trend‐topic and keyword co‐occurrence analyses.

## Data Availability

The data that support the findings of this study are available from the corresponding author upon reasonable request. The complete search query and the main analytic settings used in biblioshiny are reported in the manuscript, and the stoplist used for the thematic analyses is provided as Supporting Information.
